# Quantitative Mutation Analysis of Genes and Proteins of Major SARS-CoV-2 Variants of Concern and Interest

**DOI:** 10.3390/v15051193

**Published:** 2023-05-18

**Authors:** Fengyi Liang

**Affiliations:** Department of Anatomy, Healthy Longevity Translational Research Program, Yong Loo Lin School of Medicine, National University Health System, National University of Singapore, Singapore 117594, Singapore; antlfy@nus.edu.sg; Tel.: +65-6-5161936

**Keywords:** severe acute respiratory syndrome coronavirus 2 (SARS-CoV-2), coronavirus disease 2019 (COVID-19), mutation, variant, viral protein, antigenicity

## Abstract

Of various SARS-CoV-2 variants, some have drawn special concern or interest because of their heightened disease threat. The mutability of individual SARS-CoV-2 genes/proteins presumably varies. The present study quantified gene/protein mutations in 13 major SARS-CoV-2 variants of concern/interest, and analyzed viral protein antigenicity using bioinformatics. The results from 187 carefully perused genome clones showed significantly higher mean percent mutations in the spike, ORF8, nucleocapsid, and NSP6 than in other viral proteins. The ORF8 and spike proteins also tolerated higher maximal percent mutations. The omicron variant presented more percent mutations in the NSP6 and structural proteins, whereas the delta featured more in the ORF7a. Omicron subvariant BA.2 exhibited more mutations in ORF6, and omicron BA.4 had more in NSP1, ORF6, and ORF7b, relative to omicron BA.1. Delta subvariants AY.4 and AY.5 bore more mutations in ORF7b and ORF8 than delta B.1.617.2. Predicted antigen ratios of SARS-CoV-2 proteins significantly vary (range: 38–88%). To overcome SARS-CoV-2 immune evasion, the relatively conserved, potentially immunogenic NSP4, NSP13, NSP14, membrane, and ORF3a viral proteins may serve as more suitable targets for molecular vaccines or therapeutics than the mutation-prone NSP6, spike, ORF8, or nucleocapsid protein. Further investigation into distinct mutations of the variants/subvariants may help understand SARS-CoV-2 pathogenesis.

## 1. Introduction

As the causative pathogen for coronavirus disease 2019 (COVID-19), severe acute respiratory syndrome coronavirus 2 (SARS-CoV-2) undergoes constant mutations, resulting in the emergence of various lineages or variants of the virus since the COVID-19 outbreak [1,2,3]. The rapid evolution of the SARS-CoV-2 virus has caused new problems like repeated infections, evasion of immune protection, and lost or weakened efficacy of vaccines, therapeutics, or diagnostics [4]. The status of SARS-CoV-2 mutations is closely monitored through genomic sequencing of virus samples from infected individuals. Various large-scale analyses of SARS-CoV-2 mutations have been conducted, some focusing on specific genes/proteins of the virus, and others mainly dealing with evolutions, transmissibility, infectivity, or virulence of the variants [5,6,7,8,9,10].

SARS-CoV-2 accumulates replication errors along the course of its spread, infection, and proliferation. The variants of concern/interest of SARS-CoV-2 refer to the mutants of the virus that harbor specific combinations of viral genome mutations and have the potential of heightened disease threat due to enhanced transmission, infection, virulence, or immune escape [1,11]. Thus, among the numerous hitherto identified SARS-CoV-2 lineages, the variants of concern/interest are more clinically relevant. Epidemiologically, much has also been learned about these SARS-COV-2 variants [1,2,3,12].

The SARS-CoV-2 genome comprises approximately 30,000 nucleotides with 12 canonical genes encoding non-structural, structural, or accessory viral proteins of which functions have been mostly investigated [13,14,15,16]. It is hypothesized that susceptibility or tolerance of individual SARS-CoV-2 genes to mutations can significantly differ, although gene mutations may occur randomly. The current investigation quantified and compared mutations in genes/proteins across major SARS-CoV-2 variants of concern/interest, assessed viral protein antigenicity, and sought to distinguish mutation-prone viral genes/proteins from those more conserved. The quantitative data could hopefully deepen our understanding of the viral molecules and provide insights into more stable viral targets for vaccines that induce immune protection independent of mutations, for therapeutics that are effective against all or most variants, or for diagnostics that remain sensitive along the evolving course of the virus. Indeed, the analyses have identified better conserved, potentially immunogenic viral proteins including the nonstructural protein 4 (NSP4), NSP13, NSP14, membrane, and open reading frame 3a (ORF3a) that appeared to be more suitable as targets for vaccines, therapeutics, or diagnostics, in contrast to the highly mutative NSP6, spike, ORF8, and nucleocapsid proteins.

## 2. Materials and Methods

Initial identification of the SARS-CoV-2 variants and clones (isolates) was carried out on the Virus portal of the National Center for Biotechnology Information (NCBI, www.ncbi.nlm.nih.gov/labs/virus/vssi/#/, accessed on 3 July 2022) or NextStrain (nextstrain.org/, accessed on 3 July 2022), using the filtering by Pango lineage tool. As of the 4th of August 2022, the NCBI Virus SARS-CoV-2 data hub (www.ncbi.nlm.nih.gov/labs/virus/vssi/#/SARS-CoV-2, accessed on 4 August 2022) had 1,428,814 GenBank or RefSeq entries of complete SARS-CoV-2 genome sequences, whereas the NextStrain Latest Global SARS-CoV-2 Analysis (open data) (nextstrain.org/ncov/open/global/6m, accessed on 4 August 2022) collected information for 2701 SARS-CoV-2 genome samples classified into clades and Pango lineages. This author also applied for access to the GISAID SARS-CoV-2 database but did not receive final approval from the website. Thus, GISAID SARS-CoV-2 data could not be covered in the current analyses. 

Genome and protein sequences of SARS-CoV-2 in the GenBank or RefSeq databases were first checked online for quality and completeness. Those without obvious sequence gaps or ambiguities were downloaded from the NCBI GenBank Nucleotide (www.ncbi.nlm.nih.gov/genbank/, accessed on 4 August 2022) or RefSeq (www.ncbi.nlm.nih.gov/refseq/, accessed on 4 August 2022) databases into a project database of Unipro UGENE software package (v43.0) [17]. After importing, the genome and protein sequence data of SARS-CoV-2 variant clones were further individually inspected for precision and for the absence of ambiguity. Overall, only a minor portion of the GenBank SARS-CoV-2 entries are usable for the quantification and comparative analysis here. For example, out of the 4119 NCBI GenBank entries for the mu variant (B.1.621), only 33 have complete SARS-CoV-2 genome, and only 16 passed the stringent quality controls for inclusion in the current analysis (based on the last confirmatory searches of the NCBI Virus portal conducted on 5 August 2022). Of the 43 theta variant SARS-CoV-2 genome deposits in the NCBI GenBank, only 12 entries presented a complete genome, and 7 passed the quality controls to enter the final project database. Issues with the disqualified complete SARS-CoV-2 genome entries include sequence gaps and nucleotide or amino acid ambiguities in the genome or protein sequences. Major nucleotide ambiguities include the “N” (any nucleotide base), “S” (C or G), “R” (A or G), “Y” (C or T), “K” (G or T), “W” (A or T), and “M” (A or C). The main protein amino acid ambiguity is the “X” (any amino acid).

Variant/subvariant identities of all individual clones in the current project database were verified again by the NCBI accession numbers through NCBI Virus accession filtering (on 2 July 2022 and on 6 August 2022). Separately, we also manually verified the variant identities by comparing them with published data of typical SARS-CoV-2 variants’ mutations (Table 1) [1,2,3].

Alignment, analysis, and mutation quantification of SARS-CoV-2 variant genes and proteins were performed by using the Unipro UGENE software package (v43.0, for Microsoft Windows OS) [17]. A phylogenetic tree of 187 genomes of the SARS-CoV-2 variants of concern/interest plus the reference genome (accession number: NC_045512) was constructed by using the MEGA11 (Molecular Evolutionary Genetics Analysis version 11 Version 11.0.11, Build 11220201-x86_64), maximum likelihood statistical method, Tamura-Nei model, and bootstrap method of phylogeny test (15 replications) [18]. Codon-based Z-tests of selection of the non-synonymous/synonymous substitution (dN/dS) ratios were performed by using MEGA11 and the Nei–Gojobori (proportion) method [18]. Prediction of viral protein antigenicity of the reference SARS-CoV-2 was carried out online at the EMBOSS Antigenic webpage (www.bioinformatics.nl/cgi-bin/emboss/antigenic, accessed on 20 July 2022), using the website’s default setting. 

Data analysis, quantification, chart generation, and statistical tests (single-factor Anova, *t*-test, and F-test) were performed using Microsoft Excel. For statistics, single-factor Anova tests were first carried out to evaluate multiple groups of data. If the Anova tests reported significant (*p* < 0.05) or highly significant (*p* < 0.01) differences, the groups of data were further compared with each other by using Student’s *t*-tests to uncover the individual data groups that differed significantly or highly significantly from the others.

## 3. Results

Overall, the current project analyzed 12,516 proteins and 12,516 genes or NSP-coding nucleotide regions of 447 carefully perused genome clones of 13 SARS-CoV-2 variants of concern/interest and 5 other delta or omicron subvariants. Regarding the variants of concern/interest (Table 1), we included 5236 proteins and 5236 genes/NSP-coding nucleotide regions of 187 SARS-CoV-2 genomes (15 clones of each variant, except for the theta variant, which had only 7 clones). Of the selected three delta and four omicron subvariants, 5880 proteins and 5880 genes (or NSP-coding regions) (30 clones of each subvariant) were quantified. Mutations in the genomes, genes, and proteins of the variants/subvariants were analyzed against three NCBI GenBank/RefSeq reference SARS-CoV-2 genome clones (Table 1). As the reference genomes presented the same nucleotide sequence (except at the 3′-end poly-A tails), quantitative mutation analysis of the variants and subvariants was all conducted against the RefSeq SARS-CoV-2 reference genome (accession number: NC_045512).

**Figure 1:**
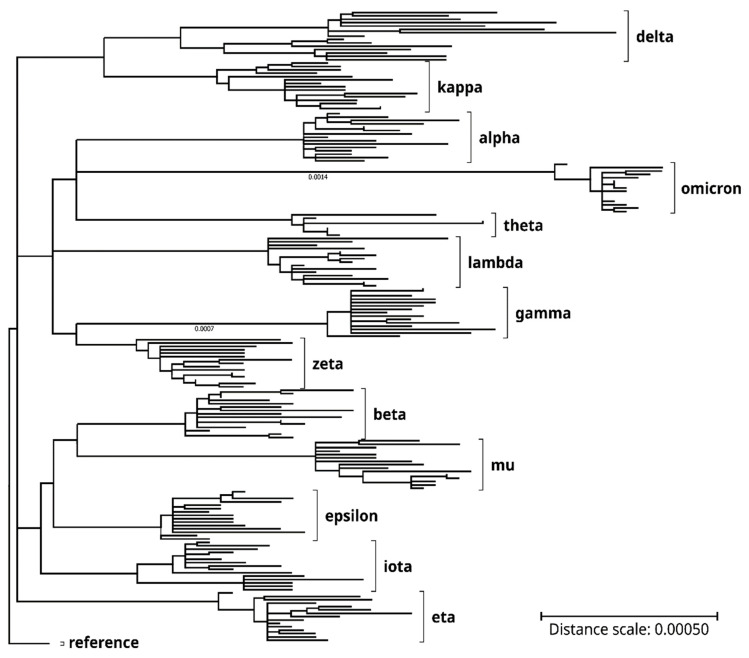
Genome phylogenetic tree of the SARS-CoV-2 clones and variants of concern/interest that were analyzed in the present study. Lengths of the tree branches represent relative phylogenetic distances between the clones and variants. Labels on the right denote the variants of concern/interest. Phylogenetic distance scale bar: 0.0005. See the Methods section for the generation of the phylogenetic tree by using the MEGA11 application software (version 11.0.11).

Genome sequences covered in the current project were identified on the NCBI Virus portal or the NextStrain website and imported from NCBI GenBank or RefSeq databases (see Methods for details). The genomes were mainly selected based on nucleotide sequence completeness (lengths > 29,550 nucleotides), sequence data quality (i.e., lack of sequence gaps, ambiguities or errors), and representativeness of mutation patterns as compared with published data in print or online [1,2,3]; [SARS-CoV-2 Variants Overview at www.ncbi.nlm.nih.gov/activ, accessed on 4 August 2022]. See Table 1 for the analyzed SARS-CoV-2 variants of concern/interest and typical mutations in each in the spike (S) and other proteins. Phylogenetic analyses of the genome and spike protein also confirmed variant identities of the respective SARS-CoV-2 clones. See Figure 1 for the genome phylogenetic tree of the 187 SARS-CoV-2 variant clones, as compared with the RefSeq reference genome. 

Relative to the reference sequence (NC_045512), the analyzed SARS-CoV-2 variants genomes here spanned from a 72-nucleotide 5′-untranslated region (UTR), across all canonical SARS-CoV-2 genes, to a 53-nucleotide 3′-UTR. Genome regions further upstream or downstream of these regions were excluded from mutation quantification to avoid potential sequencing irregularities at the extreme 5′- or 3′-end.

### 3.1. SARS-CoV-2 Genome and Gene Mutations across the Variants of Concern/Interest

**Figure 2:**
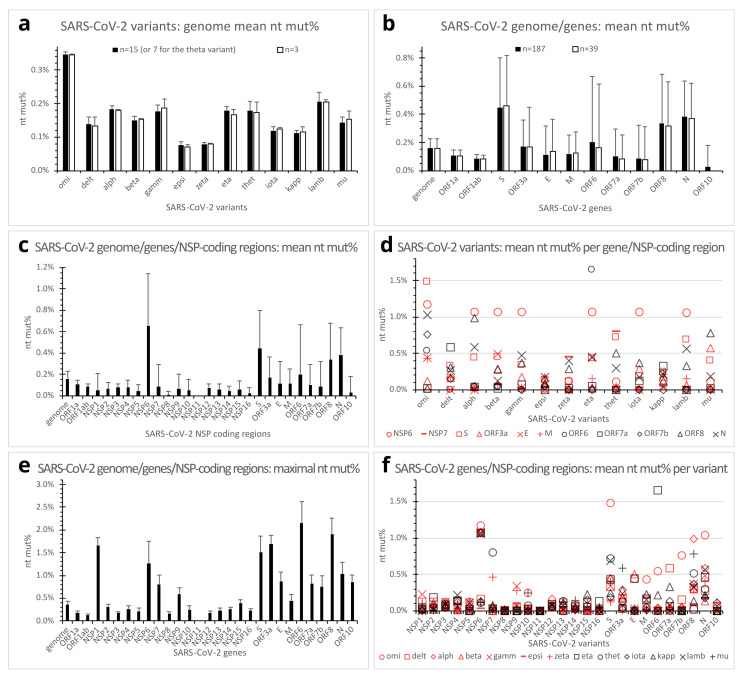
Quantitative mutations in genomes, genes, and NSP-coding regions of SARS-CoV-2 variants of concern/interest. (**a**). Mean genome nucleotide percent mutations (nt mut%) of SARS-CoV-2 variants of concern/interest. Error bars denote standard deviations. Filled bars, n = 15 (except n = 7 for the theta variant); open bars, n = 3 clones. (**b**). Mean nt mut% in SARS-CoV-2 genome and genes across SARS-CoV-2 variants of concern/interest. Error bars denote standard deviations. Filled bars, n = 187; open bars, n = 39 clones. (**c**). Mean nt mut% in SARS-CoV-2 nonstructural protein-coding regions, compared with the genome, structural, and accessory protein genes. Error bars denote standard deviations (n = 187 clones). (**d**). Dot plot showing mean nt mut% profiles of genes or NSP-coding regions of individual SARS-CoV-2 variants. Each dot represents the mean nt mut% in 15 (or 7 for the theta) in-variant clones of a gene or NSP-coding region. The genome or genes with in-variant mean nt mut% below 0.35% for all variants were omitted from the plot. (**e**). Maximal nt mut% in SARS-CoV-2 genome, genes, or NSP-coding regions out of the 187 analyzed genome clones. Error bars denote standard deviations (n = 187). (**f**). Dot plot illustrating SARS-CoV-2 variants’ mean nt mut% profiles of individual genes/NSP-coding regions. Each dot represents the mean nt mut% in a viral gene/NSP-coding region of a variant (n = 15 except n = 7 clones for the theta variant). Abbreviations: alph, alpha variant; delt, delta variant; E, envelope protein gene; epsi, epsilon variant; gamm, gamma variant; kapp, kappa variant; lamb, lambda variant; M, membrane protein gene; N, nucleocapsid protein gene; NSP, nonstructural protein gene; nt mut%, nucleotide percent mutation; omi, omicron variant; ORF, open reading frame; S, spike protein gene; thet, theta variant.

With some exceptions, such as the reference and some omicron variant clones, most in-the-same-variant (henceforth referred to as in-variant) genome clones analyzed in the present study differed from one another in mutation patterns because of additional mutation(s), back mutation(s), and so on. Of the 187 clones of 13 SARS-CoV-2 variants of concern/interest, genome nucleotide percent mutations (nt mut%) ranged from 0.064% (in the epsilon variant) to 0.356% (in the omicron variant), as compared with the reference clones. The omicron had a mean genome nt mut% of 0.347% (standard deviation (SD), 0.007%; range, 0.325% to 0.356%; n = 15 genomes) that was markedly higher than any of the other variants, whereas the delta variant had a mean genome nt mut% of 0.138% (SD, 0.022%; range, 0.102% to 0.173%; n = 15 genomes) that was at approximately the same level as the alpha, beta, gamma, theta, and mu variants (Figure 2a, filled bars).

The SARS-CoV-2 genome comprises 12 canonical genes. The ORF1a and ORF1ab genes encode the polyprotein 1a and 1ab, respectively, while the S, E, M, and N genes encode the spike (S), envelope (E), membrane (M), and nucleocapsid (N) structural proteins, respectively. The ORF3a, ORF6, ORF7a, ORF7b, ORF8, and ORF10 genes encode accessory or potential accessory proteins [19]. Comparison of mean nucleotide percent mutations across the variants of concern/interest revealed markedly different propensities of the genes for mutations. The S, N, and ORF8 genes appeared the most prone to mutations, with mean nt mut% of 0.447% (SD, 0.355%), 0.384% (SD, 0.254%), and 0.338% (SD, 0.346%) (n = 187 each), respectively. In contrast, the ORF1a and ORF1ab genes seemed more conserved (less susceptible to mutations), with low mean nt mut% of 0.106% (SD, 0.043%) and 0.087% (SD, 0.026%) (n = 187 each), respectively. The E, M, ORF7a, and ORF7b genes also exhibited relatively low mean nt mut%, whereas the ORF3a and ORF6 genes had somehow intermediate mean nt mut% of 0.169% (SD, 0.193%) and 0.201% (SD, 0.466%) (n = 187 each), respectively. Mean nt mut% was very low for the ORF10 gene (Figure 2b, filled bars).

The ORF1a and ORF1ab are overlapping long genes (13,218 and 21,291 nucleotides, respectively), encoding the ORF1a and ORF1ab polyproteins that are cleaved after translation into 11 (NSP1-11) and 15 (NSP1-NSP10, NSP12-NSP16) NSPs, respectively. Even though ORF1a and ORF1ab gave relatively low overall nt mut% values, further analysis revealed markedly different nt mut% profiles of the various NSP-coding regions. NSP6-coding region, in particular, presented the highest mean nt mut% (0.661%; SD, 0.484%; n = 187) among all the viral genes/NSP-coding regions. The other NSP-coding regions showed either no mutation (NSP11) or low-to-moderate mean nt mut% ranging from 0.008% (NSP8) to 0.088% (NSP7) (Figure 2c).

As compared per gene per variant, the mutation profile of the omicron variant appeared distinct from that of the delta variant. The former showed a much higher mean nt mut% in the S, N, ORF7b, and NSP6 genes, whereas the latter had a higher mean nt mut% in the ORF7a gene (Figure 2d,f).

Maximal nucleotide percent mutations across the sampled variants of concern/interest were also analyzed to assess the tolerance of SARS-CoV-2 genes/NSP-coding regions to mutations. Much higher maximal nt mut% (1.032–2.151%) were observed in the ORF6, ORF8, ORF3a, S, NSP6, and N genes/NSP-coding region, as compared with the ORF1a, ORF1ab, NSP12, NSP3, NSP8, and NSP11 that showed relatively low maximal nt mut% or no mutation (0.000–0.182%) (Figure 2e).

Statistical tests across different genes/NSP-coding regions confirmed the highly significant (Anova and Student’s *t*-test, *p* < 0.01) higher mean nt mut% in the NSP6, S, ORF8, and N genes, as compared with all the other genes/NSP-coding regions (n = 187 each). Mean nt mut% in the ORF3a was significantly lower than those of the NSP6, S, ORF8, and N genes, but significantly higher than most of the other genes (Anova and Student’s *t*-test, *p* < 0.01, n = 187) except the ORF6. Similarly, mean nt mut% of the ORF6 gene was significantly lower than the NSP6, S, ORF8, and N, but significantly higher than most other genes/NSP-coding regions (Anova and Student’s *t*-test, *p* < 0.01, n = 187 clones) except the ORF3a, E, and M.

Z-tests of the dN/dS substitution ratios indicated significant or highly significant positive selections (*p* < 0.05/0.01, n = 187 clones of the 13 variants of concern/interest) of the S, and M genes of the omicron variant and the N gene of the delta variant. The ORF1a and ORF1ab genes exhibited significant purifying selections (*p* < 0.05, n = 187) in the gamma and eta variants.

Prior to having the final dataset of 187 genome clones, 39 SARS-CoV-2 genome clones (3 for each of the 13 variants of concern/interest) were preliminarily analyzed for mutations. Surprisingly, results from this preliminary dataset (open bars in Figure 2a,b) showed similar mean nt mut% profiles of the genomes and genes to those derived from the finalized dataset of 187 clones (filled bars in Figure 2a,b). However, as the sample number increased, more variable maximal nt mut% in individual SARS-CoV-2 genes were observed, especially in the ORF3a, E, ORF7a, and ORF10 genes (Figure 2e).

### 3.2. SARS-CoV-2 Protein Mutations across the Variants and Subvariants

**Figure 3:**
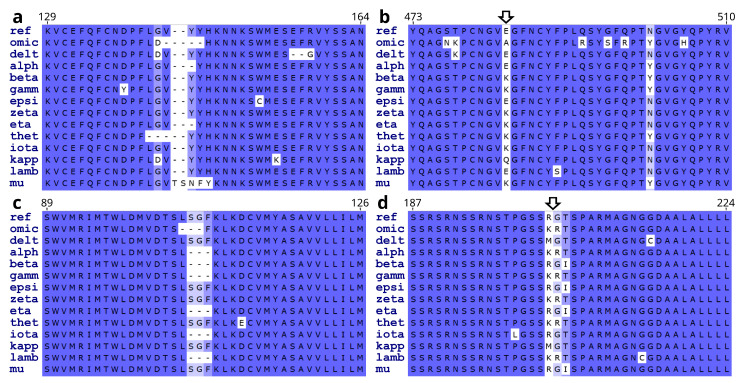
Examples of SARS-CoV-2 protein mutations across the variants of concern/interest. (**a**). Substitutions, deletions, and insertions in the S protein (near the N-terminus). (**b**). Substitutions in the S protein (in the receptor binding domain). (**c**). Deletions and substitutions in the NSP6. (**d**). Substitutions in the N protein. Arrows point to some of the mutation hotspots. Labels on the left of each panel denote the reference SARS-CoV-2 (ref) or the variants. Numbers above the panels indicate positions of the marked amino acids in the reference viral proteins. See the legend to Figure 2 for abbreviations of the SARS-CoV-2 variants.

Gene nucleotide mutations may or may not alter the amino acid residue (aa) sequences of translated proteins. In case of altered protein sequence, the results can be a substitution, deletion, insertion, protein truncation, or shift of open reading frame. Of the SARS-CoV-2 variants analyzed here, non-synonymous nucleotide mutations mostly led to point mutations (substitution, deletion, or insertion). Occasionally, nonsense mutations (causing protein truncations) or shifts of open reading frames were observed, with the latter resulting in either aa substitutions followed by protein truncation or nonstop protein extension beyond the normal stop codon. Below, point mutations of SARS-CoV-2 proteins are presented first. Results on rarer truncations and frameshift nonstop mutations follow toward the end of the section. Figure 3 shows a few examples of the substitution, deletion, or insertion point mutations in the S, N, and NSP6 proteins across the SARS-CoV-2 variants of concern/interest. Note some of the mutation hotspots (arrows).

**Figure 4:**
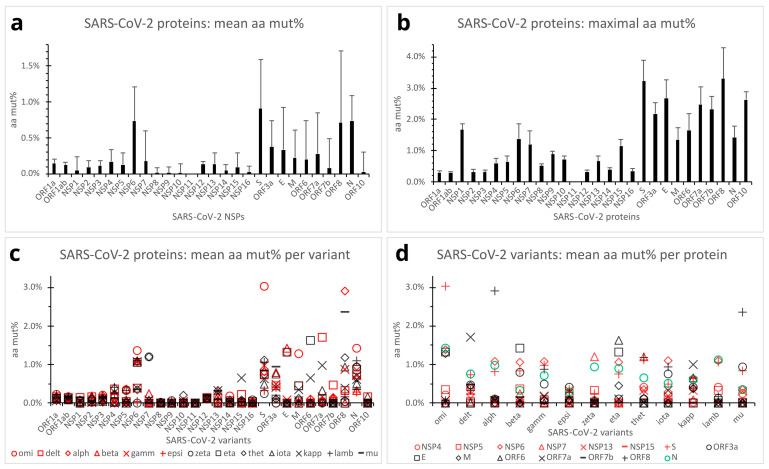
Quantification of SARS-CoV-2 protein mutations across the variants of concern/interest. (**a**). Mean aa mut% in SARS-CoV-2 proteins across the variants. Error bars denote standard deviations (n = 187 clones). (**b**). Maximal aa mut% in SARS-CoV-2 proteins across the 187 clones of the 13 variants of concern/interest. Error bars denote standard deviations (n = 187). (**c**). Dot plot comparing SARS-CoV-2 proteins by mean aa mut% profiles of the variants. Each dot represents the mean aa mut% in a viral protein of a SARS-CoV-2 variant (n = 15 clones except n = 7 for the theta variant). (**d**). Dot plot comparing the SARS-CoV-2 variants by mean aa mut% profiles of individual viral proteins (n = 15 in-variant clones except n = 7 for the theta variant). Viral proteins with in-variant mean aa mut% below 0.33% for all the variants were omitted from the plot panel (**d**). Abbreviations: aa mut%, aa percent mutation; E, envelope protein; M, membrane protein; N, nucleocapsid protein; ORF, open reading frame; S, spike protein. See the legend in Figure 2 for other abbreviations.

Overall, SARS-CoV-2 protein aa mutations across the variants of concern/interest followed the same trends as those of the gene nucleotides. The S, N, NSP6, and ORF8 proteins showed relatively high mean aa mut% (counting internal substitutions, deletions, and insertions) of 0.909% (SD, 0.683%), 0.739% (SD, 0.355%), 0.738% (SD, 0.472%), and 0.716% (SD, 0.995%; n = 187 clones of 13 variants), respectively. The ORF3a, E, and ORF7a proteins had intermediate mean aa mut% ranging from 0.278% to 0.371%. On the other hand, the ORF1a and ORF1ab polyproteins, M, ORF6, ORF7b, ORF10, and all other NSP proteins (except NSP6) exhibited no or relatively low mean aa mut% ranging from 0.000% to 0.226% (Figure 4a). Single-factor Anova and Student’s *t*-tests revealed significantly higher mean aa mut% in the S, ORF8, N, and NSP6 proteins, as compared with those of the ORF1a, ORF1ab, M, ORF6, ORF7a, ORF7b, ORF3a, E, and all other NSP proteins (*p* < 0.01) except NSP6. It should be noted that owing to the small sizes of the NSP11, E, ORF6, ORF7b, and ORF10 proteins (13, 75, 61, 43, and 38 aa residues, respectively), their mean aa mut% might statistically still be subject to significant random deviations.

Maximal aa percent mutations among the 187 SARS-CoV-2 variant clones reached high mut% of 3.306% in the ORF8 (of the alpha and theta variants) and 3.221% in the S protein (of the omicron variant) (Figure 4b). The E, ORF10, ORF7a, ORF7b, and ORF3a proteins also exhibited relatively high maximal aa mut% ranging from 2.182% to 2.667%. The NSP1, ORF6, N, NSP6, M, NSP7, and NSP15 presented maximal aa mut% ranging from 1.156% to 1.667%. Relatively low-to-moderate maximal aa mut% ranging from 0.295% to 0.885% were seen in the ORF1a, ORF1ab, and other NSP proteins (Figure 4b). Note that maximal aa mut% in the NSP11, E, ORF6, ORF7b, and ORF10 proteins might statistically still be subject to significant random errors because of the short lengths (13, 75, 61, 43, and 38 aa residues, respectively). Overall, the S and ORF8 proteins appeared capable of tolerating more mutations without a significant impact on viral survival or infectivity.

Mean aa percent mutations per viral protein per SARS-CoV-2 variant of concern/interest are presented in Figure 4c,d. As depicted, mean aa percent mutations in the S, ORF8, N, NSP6, ORF7a, and ORF6 proteins varied to a significant extent across the variants. The ORF3a, E, M, and NSP7 proteins also showed moderate to marked mean aa mut% in certain SARS-CoV-2 variants. In contrast, the ORF1a, ORF1ab, ORF10, and most other NSPs presented constantly low aa mut% across all the variants. Interestingly, the omicron variant had a much higher mean aa mut% in the structural (S, N, M, and E) and NSP6 proteins, and the delta variant had higher mean aa mut% in the ORF7a, whereas the alpha and mu variants exhibited high mean aa mut% in ORF8 (Figure 4c,d).

Besides the point mutations (above), a few frameshift or truncation mutations were observed in SARS-CoV-2 ORF8 and ORF3a proteins. These rare mutation types usually resulted in much more prominent alterations of target proteins and therefore are presented separately here to avoid distortion/bias of the point mutation data. All the alpha variant clones had a mutation that resulted in an in-frame premature stop codon and a 95mer truncation of the ORF8 C-terminus (mean aa mut%, 78.512%, n = 15). Surprisingly, the extent of mutations in ORF8 did not seriously affect the infectivity or pathogenesis of the SARS-CoV-2 alpha variant [12]. One iota variant clone had nucleotide substitution, insertion, and deletion mutations that caused a two-residue substitution followed by a nonstop extra 5mer extension of the ORF8 protein (aa mut%, 5.785%). Deletion and frameshift mutation in eight of the 15 mu variant clones produced a two-residue substitution followed by an 18mer truncation at the C-terminus of ORF3a (aa mut%, 7.273%).

**Figure 5:**
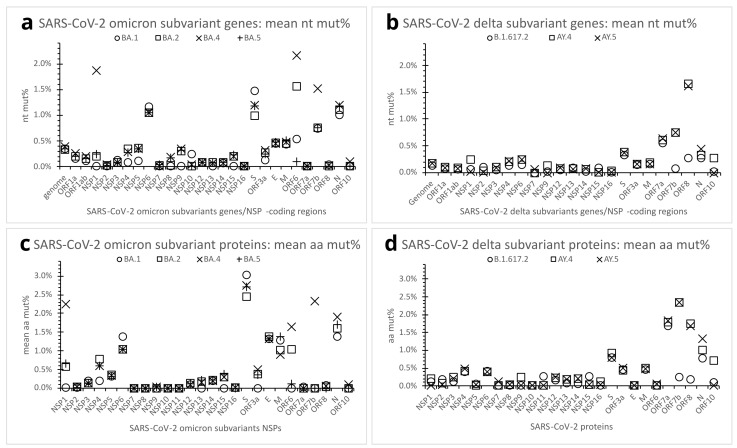
Mutation quantification of the SARS-CoV-2 omicron and delta subvariants. (**a**). Dot plot comparing nt mut% profiles of SARS-CoV-2 genome or genes (including NSP-coding regions) across the selected omicron subvariants. Each dot represents the mean nt mut% in the genome or genes of the subvariant (n = 30 in-subvariant clones). (**b**). Dot plot comparing nt mut% profiles of SARS-CoV-2 genome or genes across the selected delta subvariants. Each dot represents the mean nt mut% in the genome or genes of the subvariant (n = 30 in-subvariant clones). (**c**). Dot plot comparing aa mut% profiles of SARS-CoV-2 proteins across the selected omicron subvariants. Each dot represents the mean aa mut% in the subvariant’s protein (n = 30 in-subvariant clones). (**d**). Dot plot comparing aa mut% profiles of SARS-CoV-2 proteins across the selected delta subvariants. Each dot represents the mean aa mut% in the subvariant’s protein (n = 30 in-subvariant clones). See legends for Figure 2, Figure 3 and Figure 4 and Table 2 for abbreviations.

Some SARS-CoV-2 variants have further evolved into subvariants along the course of spreading and infection. To better understand mutation variations among subvariants, three other omicron (BA.2, BA.4, BA.5) and two other delta subvariants (AY.4, AY.5) were chosen for comparison with the supposedly root omicron subvariant BA.1 and delta subvariant B.1.617.2, respectively (Table 1 and Table 2). Thirty genome clones were sampled for each of the subvariants. The results revealed no significant difference in mean genome nt mut% in the in-variant subvariants. At the protein and gene levels, the omicron BA.2, BA.4, and BA.5 subvariants, like the BA.1, also maintained relatively high aa/nt mut% in the NSP6, S, N, E, and M proteins/genes (Figure 5a,c); the delta AY.4 and AY.5 subvariants, like the B.1.617.2, retained a relatively high mean aa/nt mut% in ORF7a (Figure 5b,d). In comparison with the BA.1, however, the omicron BA.2 subvariant showed a more marked mean aa/nt mut% in ORF6, whereas the BA.4 had higher mean aa/nt mut% in NSP1, ORF6, and ORF7b (Figure 5a,c). Relative to the B.1.617.2, the delta AY.4 and AY.5 subvariants presented higher aa/nt mut% in the ORF7b and ORF8 proteins/genes (Figure 5b,d). See Table 2 for further details of the omicron and delta subvariants’ mutations.

### 3.3. Antigen Ratios of SARS-CoV-2 Proteins

To assess the possibilities of SARS-CoV-2 proteins to elicit immune responses, we analyzed the potential antigenicity of the viral proteins using EMBOSS Antigenic. This online bioinformatic application scores potential antigenic regions of proteins using the method of Kolaskar and Tongaonkar [20,21]. The online application (https://www.bioinformatics.nl/cgi-bin/emboss/antigenic, accessed on 20 July 2022) requires an input of the target protein amino acid sequence to output a list of predicted antigenic regions of the protein. It claims a prediction accuracy of about 75% (www.bioinformatics.nl/cgi-bin/emboss/help/antigenic, accessed on 20 July 2022). Of the 26 canonical proteins of the reference SARS-CoV-2, EMBOSS Antigenic identified 436 antigenic regions with a mean antigenic region length of 18.7 aa residues (SD, 9.3; range, 7 to 55).

**Figure 6:**
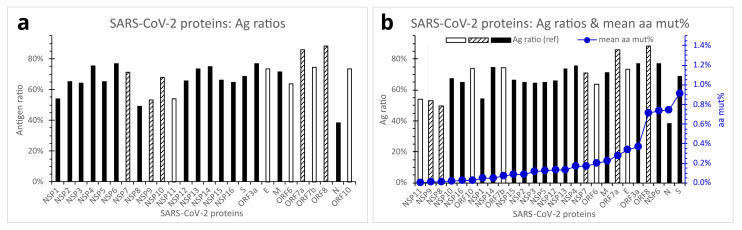
Antigen ratios of reference SARS-CoV-2 proteins in relation to mean aa mut% in the proteins across the variants of concern/interest. (**a**). Ag ratios of reference SARS-CoV-2 viral proteins. (**b**). Comparison of SARS-CoV-2 proteins with regard to Ag ratios of reference SARS-CoV-2 proteins (bars) and mean aa mut% in the viral proteins across the variants of concern/interest (closed circles). Open bars denote viral proteins measuring 11–75 aa residues in length, hatched bars denote viral proteins measuring 83–139 aa residues, and filled bars denote viral proteins measuring 180–1945 aa residues. Abbreviations: Ag, antigen; aa mut%, percent mutation of amino acid residues; E, envelope protein; M, membrane protein; N, nucleocapsid protein; NSP, nonstructural protein; ORF, open reading frame; S, spike protein.

An antigen ratio is defined as the percentage of total amino acid residue count of predicted antigenic regions divided by the amino acid residue count of the whole viral protein. Different SARS-CoV-2 proteins exhibited markedly different predicted antigen ratios ranging from 38.4% to 88.4% (Figure 6a). ORF8, ORF7a, NSP6, ORF3a, NSP4, and NSP14 presented antigen ratios equal to or above 75%. In contrast, the N, NSP8, NSP9, NSP11, and NSP1 proteins showed relatively low antigen ratios of below 55%. In particular, the N protein’s antigen ratio was only 38.4%, over 10% lower than the second lowest (NSP8, antigen ratio = 49.5%). Indeed, the predicted low Ag ratio of SARS-CoV-2 N protein concurs with previous reports of ineffective N protein of severe acute respiratory syndrome (SARS) virus in eliciting immune protection [22,23]. The S protein, which has been the target of most existing mRNA, DNA vector, or recombinant protein vaccines against SARS-CoV-2, was predicted to have an antigen ratio of 69.0%, which is lower than those of the E and M proteins, but slightly higher than those of the NSP10, NSP12, and NSP15 (Figure 6a).

**Figure 7:**
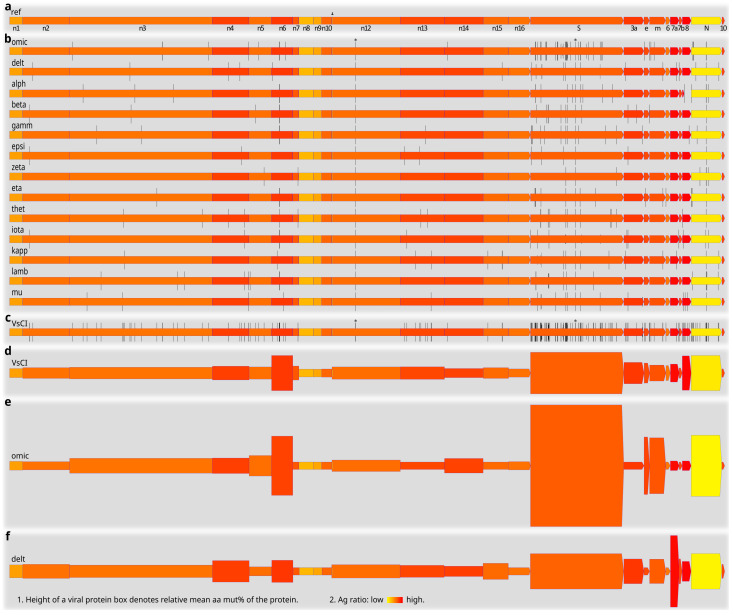
Typical mutation sites of SARS-CoV-2 variants of concern/interest, and quantification of mutations in major viral proteins and variants. (**a**). A schematic diagram showing the arrangement of SARS-CoV-2 proteins/genes in the viral genome, and the predicted antigen ratios of reference SARS-CoV-2 proteins. Yellow-colored protein boxes denote relatively low Ag ratios, whereas red-colored protein boxes denote relatively high Ag ratios. Note that non-canonical overlapping SARS-CoV-2 genes were not analyzed here or shown as individual entities. (**b**). Typical mutation sites of individual SARS-CoV-2 variants of concern/interest. Note the variable mutation profiles of the variants. Each mutation site is represented by a vertical bar. Mutations found in all clones of a variant are denoted by tall vertical bars, those found in over 80% but less than 100% of the clones by intermediate-height vertical bars, and mutations found in over 50% but less than 80% of the clones by short vertical bars. (**c**). Cumulative typical mutation sites in different SARS-CoV-2 proteins and variants of concern/interest. (**d**). Mean mut% in SARS-CoV-2 proteins [range: 0.000% (NSP11) to 0.909% (S)] across the variants of concern/interest (n = 187 clones of 13 variants) as represented by the relative heights of the viral protein boxes. (**e**). Mean mut% in SARS-CoV-2 proteins [range: 0.000% (e.g., NSP7, ORF10) to 3.043% (S)] in the omicron variant (n = 15 clones) as represented by the relative heights of the viral protein boxes. (**f**). Mean mut% in SARS-CoV-2 proteins [range: 0.000% (e.g., NSP7, NSP11) to 1.708% (ORF7a)] in the delta variant (n = 15 clones) as represented by the relative heights of the viral protein boxes. Comparison of panels (**e**,**f**) shows obvious differences between mutation profiles of the omicron and delta variants. Abbreviations: 10, ORF10; 3a, ORF3a; 6, ORF6; 7a, ORF7a; 7b, ORF7b; 8, ORF8; arrow, NSP11; e, envelope viral protein; m, membrane viral protein; n1–n16, NSP1–NSP16 viral proteins; N, nucleocapsid viral protein; S, surface viral protein; VsCI, variants of concern/interest; *, mutations found in all clones and variants of concern/interest. See Figure 2 and Figure 4 for other abbreviations.

Figure 7 presents an illustrative summary of SARS-CoV-2 protein mutations across the variants of concern/interest. The arrangement of SARS-CoV-2 proteins/genes in the viral genome is schematically shown in Figure 7a, together with predicted Ag ratios (color-coded) of the reference SARS-CoV-2 proteins. Typical viral protein mutation sites (denoted by vertical bars at the codon/aa positions in the genes/proteins) in the variants of concern/interest are shown in Figure 7b. Apart from the NSP12:P323L and S:D614G mutations found in all the variants (marked by asterisks in Figure 1b,c), most other mutation sites in the variants of concern/interest differ from each other. Further investigation of these different mutation patterns is required for a better understanding of the variable transmissibility, virulence, immune evasion, and other properties of the variants. The variable mutation propensities of different SARS-CoV-2 genes in different variants, such as the more frequent mutations in the S protein of the omicron variant and in the ORF8 of the alpha and mu variants, could already be seen by their respective typical mutation sites (Figure 7b). Superimposition of all the mutation sites in Figure 7b resulted in the cumulative typical protein mutation sites across the variants of concern/interest (Figure 7c). Overall, SARS-CoV-2 structural and accessory proteins, especially the S and ORF8, seem more densely populated by typical mutation sites, as compared with most NSPs (Figure 7c). We have summarized some important quantitative mutation findings in Figure 7d–f. Note that here the data counted not only the typical (Figure 7b,c) and other mutation sites (not shown), but also the incidence of individual mutations. The NSP6, S, ORF8, and N proteins appeared highly mutative relative to most other SARS-CoV-2 proteins (Figure 7d). In addition, different variants of concern/interest differed from each other in mutation profiles. The omicron variant, for example, had high percent mutations in the NSP6, S, E, M, and N proteins (Figure 7e), whereas the delta variant featured more mutations in the ORF7a (Figure 7f).

## 4. Discussion

The present study chose the variants of concern/interest for SARS-CoV-2 mutation analyses. In comparison with other recent reports mining genome data, this approach has the advantage of obtaining greater quantitative and comparative details from the SARS-CoV-2 whole genes/proteins, and across the variants of concern/interest. By focusing on the variants that have dominated the COVID-19 pandemic, potential biases from numerous other variants, each of which may have contributed little to the pandemic, have been largely avoided. There are, however, obvious limitations to this approach. First, by focusing on the variants of concern/interest, the study did not consider the many rarer SARS-CoV-2 variants. Second, the variants of concern/interest, as defined by the World Health Organization (www.who.int/en/activities/tracking-SARS-CoV-2-variants/, accessed on 4 August 2022), are limited in number. Thus, the current analyses are constrained by the sample size, although we made efforts to mitigate this by selecting 15 and 30 clones of each variant and subvariant, respectively. Third, it remains unclear how representative the variants of concern/interest could be of the overall SARS-CoV-2 evolution since the COVID-19 outbreak. Additionally, some may question the representativeness of the randomly chosen genome clones for a variant/subvariant. In practical terms, the number of genome clones for each SARS-CoV-2 variant was constrained by the relatively minor variants such as the theta and mu. As detailed in the Methods section, for example, even though we found many mu variant genome deposits at the NCBI Virus portal, a sizable portion of the entries there failed to meet the stringent quality control criteria and thus could not be included in the present project. Nevertheless, from the comparison between the preliminary dataset (of three clones per variant) and the final dataset (of 15 clones per variant, except for the theta variant, which had only 7 clones), it is clear that the increased sample size did not significantly alter mean mut% profiles of the viral genes (Figure 2a,b).

More importantly, the present quantification at the levels of whole genes and proteins could not differentiate mutations at different nucleotide/amino acid positions of a gene/protein. This excluded the possibility of detecting some of the mutation differences. In comparison with the omicron BA.1, for example, the omicron BA.5 subvariant showed similar mean aa percent mutation profiles across SARS-CoV-2 proteins (Figure 5c), and so seemed the omicron BF.7 subvariant. Hence, the quantitative data represent only one aspect of SARS-CoV-2 evolution. Fuller understanding of the SARS-CoV-2 variants would require the integration of data from other aspects (e.g., Table 1 and Table 2, Figure 7b,c).

Since the COVID-19 outbreak, SARS-CoV-2 evolution has been closely monitored, and many virus variants have been uncovered. Different aspects of SARS-CoV-2 mutations have been investigated, including functional constraints, adaptations, molecular variations of the genes/proteins, and clinical or epidemiological consequences of different mutations [5,6,7,8,9,10]. Overall, the present findings agree with previous data on the trend and mutation susceptibility of different SARS-CoV-2 proteins/genes [5,24]. Concerning the SARS-CoV-2 structural proteins, for example, Das and Roy (2021) reported the E and M being relatively more stable than the S and N genes/proteins [7]. Experimentally, the S protein gene has been shown to accumulate 5-times more mutations than the average of the SARS-CoV-2 genome [24]. Another study reported a low mutation rate of NSP10, consistent with the current findings [6].

There are also discrepancies between the present and previous data. In contrast to the low-level mutations in ORF1ab shown here, the viral gene/polyprotein has previously been reported as one of the most mutative in SARS-CoV-2 isolates from India [25]. Some disagreements could be due to using different data collection methods, analysis, or quantification. The current study analyzed only the variants of concern/interest that are of more clinical relevance. In contrast, many previous reports used genome sequencing data encompassing hundreds of variants from diverse geographic origins [5]. Other analyses considered only specific variants identified from specific regions [25,26]. Some reports counted only amino acid residue substitutions [7], whereas the present data also included aa deletions and insertions. More importantly, the mutation profiles of SARS-CoV-2 genes/proteins change rapidly along the course of the COVID-19 pandemic. It is only natural that the current genome and protein mutation profiles differ from previous reports. For example, the emergence of the omicron variant and subvariants has markedly altered the mutation patterns across SARS-CoV-2 genes/proteins. This trend will likely continue as long as the COVID-19 pandemic persists [9].

Mutations of the SARS-CoV-2 genome may result in altered biochemical properties and functions of proteins/genes that could in turn lead to changes in viral survival, infectivity, pathogenesis, or immune evasion [27,28,29,30,31,32]. Mutations may also cause drifts in how the virus recognizes the host cell receptor [33,34]. So far, the implications of most SARS-CoV-2 protein mutations are unclear, but the consequences of S protein variations, particularly those related to receptor binding, are beginning to be elucidated. For example, S:D614G mutation has been shown to alter the conformation of the S protein receptor binding domain (RBD) and increase the virus’s accessibility to the hACE2 receptor [35]. S protein mutations in the RBD may also affect RBD-ACE2 affinity/interaction [36]. S protein mutations might also enable the virus to evade host immune protection [37,38]. Those previous findings are corroborated by the current data showing a much higher percent mutation in the S protein of the omicron variant that is known to be more contagious and transmissible. Among mutations in other SARS-CoV-2 proteins, NSP12:P323L (ORF1ab:P4715L) has been implicated in changing the virus’s pathogenicity or transmissibility [39,40]. Some mutations in the N protein are known to increase virus replication [41]. Of particular interest with regard to the present study is the ORF7a protein that showed a prominently higher percent mutation in the delta variant. It awaits future studies to clarify whether this mutated ORF7a might be related to the delta variant’s elevated virulence [42,43,44,45]. Previous studies have indeed shown diverse important roles of ORF7a, such as immune modulation/evasion, virus-host interaction, protein trafficking, inhibition of cellular translation, and apoptosis of infected cells [13,14,15,16]. Further experimental studies are needed to validate or reject this speculation.

For the development of vaccines, diagnostics, and therapeutics against a virus, several aspects must be considered in selecting an appropriate viral protein, gene, or component as a target. An important requirement would probably be the target viral protein’s relatively high Ag ratio to ensure effective immune responses. High susceptibility and high tolerance of the target viral gene/protein to mutations should probably be avoided, to minimize the possibility of immune evasion, drug resistance, or false negative diagnosis. In Figure 6b, the mean aa percent mutations in various SARS-CoV-2 proteins across the variants of concern/interest are presented, in ascending order, against Ag ratios of the same viral proteins. As shown, the S, ORF8, N, and NSP6 proteins of SARS-CoV-2 are seemingly not perfect targets for vaccines, therapeutics, or diagnostic antibodies because of their high mutability. The N protein has the additional drawback of possessing low antigenicity.

To date, however, the S protein has been the major target of various mRNA, vector DNA, and recombinant protein vaccines against SARS-CoV-2 [46,47,48]. This choice was entirely justified at the beginning of the COVID-19 pandemic when there was no data on the mutability of SARS-CoV-2 proteins. The choice was also supported by the surface location of the S protein on the viral particle and by previous findings in SARS and MERS (middle east respiratory syndrome) viruses that confirmed the efficacy of the S protein in eliciting protective immune responses [49,50,51,52]. However, the emergence of the omicron variant has shed new light on SARS-CoV-2 mutations and revealed the highly mutative nature of the S protein. The observed reduction in the efficacy of current S protein-targeting vaccines against infection by the omicron variant/subvariants has clearly exposed the vulnerability of such vaccines to immune evasion by specific SARS-CoV-2 variants [4]. Hence, we might need to adjust our strategy and explore novel approaches for developing vaccines, therapeutics, and diagnostics. For alternative and more conserved viral targets of SARS-CoV-2 vaccines, therapeutics, or diagnostics, the NSP4, NSP13, NSP14, ORF3a, and M proteins appear to be suitable candidates, given their low-to-moderate mutability and reasonably high antigenicity. Indeed, sera from individuals after COVID-19 infection display immune reactions against not only the S protein but also the M, N, NSP3, NSP4, ORF3a, ORF8, and other viral proteins [53]. In experimental animals, a potential vaccine co-expressing the M and N proteins conveyed effective protection against weight loss and severe lung pathology after SARS-CoV-2 infection [54]. Of course, there are other important considerations in selecting vaccine or drug targets, and only clinical trials can validate the actual effect of a vaccine or therapeutic agent.

## Figures and Tables

**Table 1:** Major point mutations in proteins of SARS-CoV-2 variants of concern/interest.

Variants	In S Protein	In NSPs	In Other Proteins
Omicron (ο) (BA.1)	S: A67V, HV69--, T95I, GVYY142D---, NL211-I, ins214EPE, G339D, S371L, S373P, S375F, *K417N*, *N440K, G446S*, ST477NK, E484A, Q493R, G496S, Q498R, N501Y, *Y505H*, T547K, D614G, H655Y, N679K, P681H, *N764K*, D796Y, N856K, Q954H, N969K, L981F	NSP3: K38R, *SL1265-I*, A1892T; NSP4: T492I; NSP5: P132H; NSP6: LSG105---, I189V; NSP12: P323L; NSP14: I42V	E: T9I; M: *D3G, Q19E*, A63T; N: P13L, *ERS31--*-, RG203KR
Delta (δ) (B.1.617.2)	S: T19R, *K77T*, *EFR156G--*, L452R, T478K, D614G, P681R, *D950N*	NSP2: P129L; NSP3: *P822L, *NSP4: *D217N, F375S*; NSP6: *H11Q*; NSP12: P323L; NSP15: *K259R*	ORF3a: S26L; M: I82T; ORF7a: *V82A, T120I*; N: D63G, *R203M*, *D377Y*
Alpha (α) (B.1.1.7)	S: HV69--, Y144-, N501Y, A570D, D614G, P681H, T716I, S982A, D1118H	NSP3: T183I, A890D, I1412T; NSP6: SGF106---; NSP12: P323L	ORF8: Q27 *, R52I, Y73C; N: D3L, RG203KR/KP, S235F
Beta (β) (B.1.351)	S: D80A, D215G, LLA241---, K417N, E484K, N501Y, D614G, A701V	NSP2: T85I; NSP3: K837N; NSP5: K90R; NSP6: SGF106---; NSP12: P323L	ORF3a: Q57H, *S171L*; E: P71L; N: T205I
Gamma (γ) (P.1)	S: L18F, T20N, P26S, D138Y, R190S, K417T, E484K, N501Y, D614G, H655Y, T1027I, V1176F	NSP3: S370L, K977Q; NSP6: *SGF106---*; NSP12: P323L; NSP13: E341D	ORF3a: S253P; ORF8: *E92K; *N: P80R, *RG203KR*
Epsilon (ε) (B.1.427)	S: S13I, W152C, L452R, D614G	NSP2: *T85I*; NSP4: *S395T*; NSP12: *P323L*; NSP13: *P53L*, D260Y	ORF3a: Q57H; N: T205I
Zeta (ζ) (P.2)	S: E484K, D614G, V1176F	NSP5: L205V; NSP7: L71F; NSP12: P323L	N: A119S, RG203KR, *M234I*
Eta (η) (B.1.525)	S: *Q52R*, A67V, HV69--, Y144-, E484K, D614G, Q677H, F888L	NSP3: T1189I; NSP6: SGF106---; NSP12: P323F	E: L21F; M: I82T; ORF6: F2-; N: *SD2-Y*, A12G, T205I
Theta (θ) (P.3)	S: LGV141---, LA242--, Y265C, E484K, N501Y, D614G, P681H, E1092K, H1101Y, V1176F	NSP3: D736G, S1807F; NSP4: D217N, *L438P*; NSP6: D112E; NSP7: L71F; NSP12: P323L; NSP13: L280F, A368V	ORF8: K2Q; N: RG203KR
Iota (ι) (B.1.526)	S: L5F, T95I, D253G, *S477N*, *E484K*, D614G, *A701V*, *Q957R*	NSP2: T85I; NSP4: L438P; NSP6: *SGF106---*; NSP12: P323L; NSP13: Q88H	ORF3a: P42L, Q57H; ORF7a: *L116F; *ORF8: T11I; N: *P199L*, *M234I*
Kappa (κ) (B.1.617.1)	S: *T95I*, *G142D*, *E154K*, L452R, E484Q, D614G, P681R, Q1071H	NSP3: T749I; NSP6: T77A; NSP12: P323L; NSP13: *G206C*, M429I; NSP15: *P65S*, K259R, *S261A*	ORF3a: S26L; M: *I82S*; ORF7a: V82A; N: R203M, D377Y
Lambda (λ) (C.37)	S: GT75VI, *RSYLTPGD246-------N*, L452Q, F490S, D614G, T859N	NSP3: T428I, P1469S, F1569V; NSP4: L438P, T492I; NSP5: G15S; NSP6: SGF106---; NSP12: P323L	N: P13L, RG203KR, G214C, *T366I*
Mu (μ) (B.1.621)	S: T95I, *YY144TSN-*, R346K, E484K, N501Y, D614G, P681H, D950N	NSP3: T237A, T720I; NSP4: T492I; NSP6: Q160R; NSP12: P323L; NSP13: P419S	ORF3a: Q57H, VNP256IQ *; ORF8: *T11K*, P38S, S67F; N: T205I
Reference sequences (NCBI accession numbers: NC_045512, MN996528, MN908947): no mutation			

(1) Point mutations are denoted first by the one-letter code(s) of the wild-type aa(s) followed by the numerical position of the (first) mutated aa and then the substitution aa(s), deletion (“-”), or stop codon (“*”). In case of insertion (“ins”), the number following “ins” denotes the numerical position of the aa preceding the insertion. (2) *Italics*: Mutations that are found in over 80% of the variant clones, but not all; *italics and gray*: Mutations that are found in over 50% of the variant clones, but below 80%; highlighted mutations are found in all variant clones. (3) Viral protein names are underlined; different proteins are separated by a semicolon (;). E, envelope protein; M, membrane protein; N, nucleocapsid protein; ORF, open reading frame; S, spike protein.

**Table 2:** Major point mutations in selected SARS-CoV-2 omicron and delta subvariant proteins.

Subvariants	In S Protein	In NSPs	In Other Proteins
Omicron BA.1	S: A67V, HV69--, T95I, GVYY142D---, NL211-I, ins214EPE, G339D, *S371L*, S373P, S375F, *K417N*, *N440K, G446S*, ST477NK, E484A, *Q493R*, *G496S*, *Q498R*, *N501Y*, *Y505H*, T547K, D614G, H655Y, N679K, P681H, *N764K*, D796Y, N856K, *Q954H*, N969K, L981F	NSP3: K38R, *SL1265-I*, A1892T; NSP4: T492I; NSP5: P132H; NSP6: LSG105---, I189V; NSP12: P323L; NSP14: I42V	E: T9I; M: *D3G, Q19E*, A63T; N: P13L, *ERS31--*-, *RG203KR*
Omicron BA.2	S: T19I, LPPA24---S, G142D, V213G, G339D, S371F, S373P, ST375FA, D405N, R408S, K417N, *N440K*, ST477NK, E484A, Q493R, Q498R, N501Y, Y505H, D614G, H655Y, N679K, P681H, N764K, D796Y, Q954H, N969K	NSP1: S135R; NSP3: T24I, G489S; NSP4: *L264F*, T327I, L438F, T492I; NSP5: P132H; NSP6: SGF106---; NSP12: P323L; NSP13: R392C; NSP14: I42V; NSP15: T112I	ORF3a: T223I; E: T9I; M: Q19E, A63T; ORF6: *D61L*; N: P13L, ERS31---, RG203KR, *S413R*
Omicron BA.4	S: T19I, LPPA24---S, HV69--, G142D, V213G, G339D, S371F, S373P, ST375FA, D405N, R408S, K417N, *N440K*, *L452R*, ST477NK, E484A, F486V, Q498R, N501Y, Y505H, D614G, H655Y, *N658S*, N679K, P681H, N764K, D796Y, Q954H, N969K	NSP1: S135R, KSF141---; NSP3: T24I, G489S; NSP4: *L264F*, T327I, T492I; NSP5: P132H; NSP6: SGF106---; NSP12: P323L; NSP13: R392C; NSP14: I42V; NSP15: T112I	ORF3a: *T223I*; E: T9I; M: Q19E, A63T; ORF6: D61L; ORF7b: L11F; N: P13L, ERS31---, P151S, RG203KR, S413R
Omicron BA.5	S: T19I, LPPA24---S, HV69--, G142D, V213G, G339D, S371F, S373P, ST375FA, D405N, R408S, K417N, N440K, L452R, ST477NK, E484A, F486V, Q498R, N501Y, Y505H, D614G, H655Y, N679K, P681H, N764K, D796Y, Q954H, N969K	NSP1: S135R; NSP3: T24I, G489S; NSP4: *L264F*, T327I, T492I; NSP5: P132H; NSP6: SGF106---; NSP12: P323L; NSP13: R392C; NSP14: I42V; NSP15: T112I	ORF3a: *T223I*; E: T9I; M: D3N, Q19E, A63T; N: P13L, ERS31---, RG203KR, S413R
Delta B.1.617.2	S: T19R, *K77T*, *EFR156G--*, L452R, T478K, D614G, P681R, D950N	NSP2: P129L; NSP3: *P822L*; NSP4: *D217N, F375S*; NSP6: *H11Q*; NSP12: P323L; NSP15: *K259R*	ORF3a: S26L; M: I82T; ORF7a: *V82A, T120I*; N: D63G, *R203M*, *D377Y*
Delta AY.4	S: T19R, T95I, *G142D*, *E156G*, FR157--, L452R, T478K, D614G, P681R, D950N,	NSP3: A488S, P1228L, P1469S, A1711V; NSP4: V167L, T492I; NSP6: T77A; NSP12: P323L, G671S; NSP13: P77L; NSP14: A394V	ORF3a: S26L; M: I82T; ORF7a: V82A, T120I; ORF7b: T40I; ORF8: DF119--; N: D63G, R203M, G215C, D377Y
Delta AY.5	S: T19R, *G142D*, *E156G*, FR157--, L452R, T478K, D614G, P681R, D950N,	NSP3: A488S, P1228L, P1469S; NSP4: V167L, T492I; NSP6: T77A; NSP12: P323L, G671S; NSP13: P77L; NSP14: A394V	ORF3a: S26L; M: I82T; ORF7a: V82A, *T120I*; ORF7b: T40I; ORF8: DF119--; N: D63G, R203M, G215C, S327L, D377Y

(1) Point mutations are denoted first by the one-letter code(s) of the wild-type aa(s) followed by the numerical position of the (first) mutated aa and then the substitution aa(s) or deletion (“-”). In case of an insertion mutation (“ins”), the number following “ins” denotes the numerical position of the aa preceding the insertion. (2) *Italics*, mutations that are found in more than 80% of the subvariant clones, but not all; highlighted, mutations that are found in all subvariant clones. (3) Viral protein names are underlined; different proteins are separated by a semicolon (;). E, envelope protein; M, membrane protein; N, nucleocapsid protein; ORF, open reading frame; S, spike protein.

## Data Availability

Further details of the analyzed SARS-CoV-2 variants clones, together NCBI GenBank/Refseq accession numbers of each, variant belonging/Pango lineage, countries of origin, dates of sample collection and data submission to NCBI, submitters and so on are available from the author upon request.
